# Cluster randomized trial assessing the effects of rapid ethical assessment on informed consent comprehension in a low-resource setting

**DOI:** 10.1186/s12910-016-0127-z

**Published:** 2016-07-12

**Authors:** Adamu Addissie, Serebe Abay, Yeweyenhareg Feleke, Melanie Newport, Bobbie Farsides, Gail Davey

**Affiliations:** Brighton and Sussex Medical School, Brighton, UK; Addis Ababa University, Addis Ababa, Ethiopia; Ethiopian Public Health Institute, Addis Ababa, Ethiopia

**Keywords:** Informed consent process, Informed consent comprehension, Quality of informed consent, Rapid Ethical Assessment (REA), Ethiopia

## Abstract

**Background:**

Maximizing comprehension is a major challenge for informed consent processes in low-literacy and resource-limited settings. Application of rapid qualitative assessments to improve the informed consent process is increasingly considered useful. This study assessed the effects of Rapid Ethical Assessment (REA) on comprehension, retention and quality of the informed consent process.

**Methods:**

A cluster randomized trial was conducted among participants of HPV sero-prevalence study in two districts of Northern Ethiopia, in 2013. A total of 300 study participants, 150 in the intervention and 150 in the control group, were included in the study. For the intervention group, the informed consent process was designed with further revisions based on REA findings. Informed consent comprehension levels and quality of the consent process were measured using the Modular Informed Consent Comprehension Assessment (MICCA) and Quality of Informed Consent (QuIC) process assessment tools, respectively.

**Result:**

Study recruitment rates were 88.7 % and 80.7 % (*p* = 0.05), while study retention rates were 85.7 % and 70.3 % (*p* < 0.005) for the intervention and control groups respectively. Overall, the mean informed consent comprehension scores for the intervention and control groups were 73.1 % and 45.2 %, respectively, with a mean difference in comprehension score of 27.9 % (95 % CI 24.0 % - 33.4 %; *p* < 0.001,). Mean scores for quality of informed consent for the intervention and control groups were 89.1 % and 78.5 %, respectively, with a mean difference of 10.5 % (95 % CI 6.8 -14.2 %; *p* < 0.001).

**Conclusion:**

Levels of informed consent comprehension, quality of the consent process, study recruitment and retention rates were significantly improved in the intervention group. We recommend REA as a potential modality to improve informed consent comprehension and quality of informed consent process in low resource settings.

**Electronic supplementary material:**

The online version of this article (doi:10.1186/s12910-016-0127-z) contains supplementary material, which is available to authorized users.

## Background

Informed consent comprehension is an important determinant of study compliance and retention [1]. Quality in the informed consent process include adequacy of the information provided; understand-ability of the purpose, benefits and risks of the research; distinction between research and clinical care; voluntariness of participation; recall of signing a consent document; and satisfaction with the consent process as well as the levels of recall and understanding by study participants [2]. Important parameters in monitoring the quality of research implementation in addition to levels of study recruitment include levels recall and understanding of the informed consent information by study participants [3–7]. To this effect, informed consent documents may require to be adapted to the local culture and the educational level of the population, which have been shown to strengthen research efforts, through improved recruitment and retention of participants who better understand their roles and responsibilities [8–10].

Rapid assessment techniques such as Rapid Ethical Assessment (REA) have been documented to play an important role in improving informed consent process for medical research in developing countries by providing better understanding of research contexts [11–14]. REA is a brief (an average of 6 weeks long) qualitative intervention designed to map the ethical terrain of the research setting prior to a research team starts recruiting participants with the purpose of connecting ethical principles to contexts and realities on the ground. REA attempts to discover, describe and respond to the ethical issues specific to a particular research setting, and help researchers to address the issues that genuinely matter to proposed study participants and their community. REA methodology involves interviews, focused discussion and observation conducted among key stakeholders to inform the design of the particular research project. Its findings are utilised to inform and guide the research consent process; ranging from the conception and development of the consent form, to the way consent is obtained. It has been documented that REA is considered relevant and of utmost use for researchers in low income settings [15]. However, the effects of REA on study participants’ level of comprehension, recruitment and retention and quality of informed consent have not been documented.

The current study investigated effects of REA on informed consent comprehension, study compliance and quality of informed consent in a low-income setting. We measured consent information comprehension level, recruitment rate, retention rate and quality-of-informed consent among the participants enrolled into another research: “*HPV-Subtype Prevalence Study*” [16] in the Tigray Regional State of Northern Ethiopia. The HPV study was identified for the REA intervention due to potential ethical issues anticipated in the study.

## Methods

### Study design

A cluster randomised controlled trial was conducted among participants of an HPV Sero-prevalence study in Northern Ethiopia, from July 8 to August 23, 2013. The intervention for the trial was conducting REA and subsequent revision of the informed consent's content and processes based on the REA findings. The intervention and control groups were compared for levels of comprehension, recruitment, retention (compliance) and perceived qualities of the consent process two weeks after initial consent. More description on the measurements is given in the data collection procedures section below.

### Source and study population

Pregnant women between 18-45 years attending ANC follow-up in four selected health facilities and who were targeted for the HPV sero-prevalence study were both the source and study population for this comprehension assessment. Those with communication challenge, severe illness, gestational-age greater than 34 weeks, or those who were not willing to return after two weeks for the follow-up interview were excluded.

### Sample size determination

Sample size was determined using the sample size formula for double-population proportion -

$$ \frac{\mathrm{n} = {\mathrm{P}}_1\Big(1 - {\mathrm{P}}_{1\Big) + }{\mathrm{P}}_2\left(1-{\mathrm{P}}_2\right)\ \mathrm{X}\ \mathrm{f}\ \left(\upalpha,\ \upbeta \right)}{{\left({\mathrm{P}}_2 - {\mathrm{P}}_1\right)}^2} $$

where ;

n : number of participants per group

p_1_ : expected levels of comprehension in the control group

p_2_ : expected levels of comprehension in the intervention group

α : degree of error

β : power of the study

Level of comprehension (P_1_) for the control group was estimated based on an earlier study (hence P_1_ = 0.75) [3]. We assumed an improvement in the level of comprehension of 13 % (P_2_ = 0.88) for the intervention group with 80 % power (β = 0.80), and 95 % confidence interval (α = 0.05) (hence A(α, β) = 7.85). After adjusting for 10 % non-response rate, the total sample size required was 300 participants with a 1:1 ratio between intervention and control participants (i.e. 150 in each group).

### Sampling procedure

**Fig. 1:**
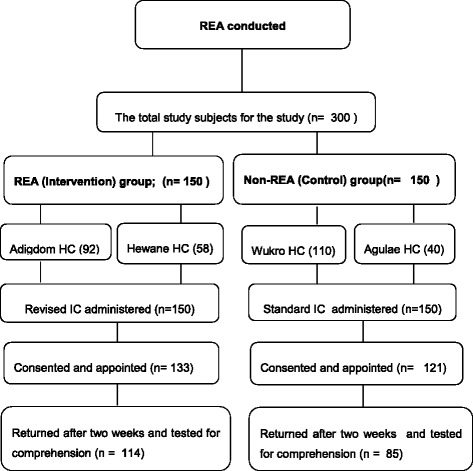
Flow-Chart showing the sampling procedures for the study. Illustrates the sampling procedures of the study and the steps followed in the recruitment of study subject for the study. Recruitment of the study participants followed the REA (Rapid Ethical Assessment) which was conducted at the very beginning. The study participant were in total 300, with 150 in the intervention and 150 in the control arms. They were selected from two health centres in both instances and the number selected were proportional to the turn-out of patients in each health institution

Two *woredas* (districts) targeted for the HPV sero-survey were selected purposively based on comparability. The selected *woredas* were randomly assigned using a lottery method to either intervention or control. Health centres within each of the *woredas* were shortlisted based on: availability of Ante Natal Care (ANC) service provision, laboratory services, and appropriate professionals to collect and handle specimen and flow of adequate number of ANC clients. Two health centres from each *woredas* (i.e. a total of four health centres), were randomly selected from the shortlisted health centres (Fig. 1). Sample size was proportionally allocated to the health facilities based on reported flow of ANC clients in the previous one year reported by the respective health bureau at the district level.

### Data collection procedures, instruments and variables

REA conducted at the beginning of recruitment identified a number of ethical considerations. Based on these findings revisions were suggested on (a) the consent form and information sheet and (b) the consent process and procedures (Table 1). Accordingly the IRB-approved consent form (Additional file 1) was revised for the intervention group (Additional file 2). The revisions made were: use of additional local terminologies, concrete explanations of study information with examples, and contextual clues based on the REA findings. Consent information was provided by reading the information sheet to participants in both groups. In the intervention group, this was accompanied by additional narratives which were attached to the consent document based on the issues identified by REA. Accordingly participants were asked whether they had any questions after reading each section of the form, and once more after the end of the entire reading. Consent in the control group, was obtained by signing on the consent form, but verbally in the intervention group with data collectors signing as witnesses. Sentences and paragraphs in the revised version of informed consent form were relatively short, and the words chosen were more familiar and local. The major characteristics of the two versions are summarized in Table 1.

**Table 1:** Comparison of 'standard' and 'revised' versions of information sheet, consent forms and procedures

Consent form information	Revised version	Standard version
Font, style	12, power geez uni-code 1	12, power geez uni-code 1
Language	Tigrigna	Tigrigna
Average words per sentence	12.4	16.3
Average words per paragraph	67.1	97.8
Average sentences per paragraph	5.6	7.6
Total words	604	587
Total number of paragraphs	9	6
Number of pages	Two and a half	Two
Average time taken (minutes)	Mean = 10 ; Median = 10	Mean = 9.6 ; Median = 10
Used narrative explanations?	Yes	No
Invited questions on unclear ideas or concepts	Yes. Written or included in the consent form.	Yes. Orally
Type of consent seeking	Verbal consent	Signed document
Number of days between consent taking and comprehension assessment	Mean =12 days Median =12 days	Mean = 11.9 days Median = 12 days

Level of comprehension of informed consent, and the quality of the consent process were assessed two weeks after consent had been taken. We used Modular Informed Consent Comprehension Assessment (MICCA) and Brief Investigator Questionnaire (BIQ) [17] to measure informed consent comprehension of participants; and Quality of Informed Consent (QuIC) [18] to assess quality of the informed consent process. The MICCA and BIQ instruments for our assessment were developed by adopting the existing generic tools (Additional file 3). Both tools have the flexibility to test comprehension specific to a particular trial, yet can be utilized across a variety of trials. MICCA measures comprehension by incorporating both generic and trial-specified approaches. Psychometric evidence suggests that MICCA can be utilized in various trial settings and can produce reliable and valid scores [17].

Comprehension was assessed as a function of understanding and recall. Understanding refers to the concepts explained in the consent process such as purpose, benefits and risk. Recall refers to issues that needed to be memorized such as date of appointment, and specific procedures such as signature, copy of consent given or not [19] (Table 2). A total of 25 questions were used to assess informed consent comprehension of participants; 13 questions were used to assess levels of understanding and 12 questions to asses levels of recall. Test items consisted of both generic and trial specific questions (Additional file 3). Of the 25 test items, 14 were generic test items that appeared on each version of MICCA, 6 were trial-specific test items which were generated based on responses to BIQ and the remaining 5 were trial-specific test items in which the response options for each of the test items are generated based on response to BIQ. QuIC consisted of 13 subjective questions on the perceived quality of the informed consent process (Additional file 3). Each correct response was scored as 1, while each incorrect response was scored 0. For multiple choices with more than one correct response a score value of 1 was given for each possible answer. All the correct answers for understanding and recall were separately summed and calculated out of hundred.

**Table 2:** Operational Definitions

Operational definitions Comprehension Level: the level of participant understanding and recall of the information they were given through the consent document. Assessment is based on the Comprehension Test administered two weeks into the study. Comprehension was categorized into three levels [high :75 % or greater; medium: between 50 % and 75 %; low: 50 % or less ] adapted from earlier studies [3]. Its components included Recall and Understanding. Recall: success in selecting or remembering the correct statement Understanding: correctness of interpretations of statements presented Study Recruitment rate: Recruitment rate is the proportion (percentage) of participants who voluntarily decide to participate in the study out of those who were approached to take part. Study Compliance (or Retention) rate: Measures compliance to follow-up and appointments as directed in the visit before. Compliance rate refers to the proportion (percentage) of participant that attended their appointment at the specified time divided by the number of participants who were given an appointment and agreed to come. Quality of informed consent process assessment: a measure of the quality of consent process concerning information adequacy, participant perception and satisfaction of the process, and the situation of decision making during the recruitment process. Scoring levels were the same as for comprehension.	

MICCA, BIQ and QuIC-based questionnaires for the study were initially prepared in English (see Additional file 3) and then translated to *Tigrigna*, the local language. The *Tigrigna* version was translated back to English to check for consistency of meaning. Data collectors who recruited participants and obtained initial consent were nurses from the respective health centres, who had been trained for the study. Comprehension assessment was done by data collectors who had been trained on the use of the assessment tools. Data collectors were supervised by public health experts with postgraduate qualifications.

### Data analysis

Data were pre-coded and entered into Epi-Info version 3.5.1. Data were cleaned and edited using simple frequencies and cross tabulation and exported to SPSS Version 16 for further analysis. Descriptive statistics were generated for describing the study population in relation to relevant socio-demographic variables. Regression analysis were done for comparative analysis and assessing association between the major outcome variables and associated factors such as educational status, occupation and previous participation in research. Risk ratios with 95 % confidence interval were used to determine levels of significance. Multivariate logistic regression model was used to control for confounders and adjust the measures of association.

## Results

### REA findings

Rapid Ethical Assessment conducted at the beginning of recruitment identified a number of ethical considerations, based on which the consent form and process for the intervention group were revised. Major REA findings included low awareness of the concept of research (therapeutic misconception), presence of indigenous descriptions and terminologies for technical disease-related terms; concerns about biological samples; major discomfort around signing a consent form; discomfort with male enumerators; suspicions around the study selection criteria; concerns with outsiders coming to the community with incentives; and concerns over test results and their implications. These findings dictated the need for further elaboration on some of the components of the research, for extended discussion on the issues identified, and for changes in the consenting mechanism.

### Socio-demographic and economic characteristics of study participants

A total of 300 pregnant women were approached for the study, each half from control and intervention locations. Of the 254 who consented (133 from intervention and 121 from control sites), 199 (114 from intervention and 85 from control site) also came to the follow-up visit after two weeks and participated in the comprehension assessment.

The mean age (±2 SD) of the participants who complied with the follow-up appointment was 27.3 (+/-6.3) years. Majority (83.4 %) were married; from the Tigre ethnic group (99.5 %); Orthodox by religion (86.4 %); able to read and write (70.8 %); and had a monthly family income of >1000 Ethiopian Birr ETB) (61.8 %) which is equivalent to 50 USD. For most participants (72.9 %), it was the first time they had participated in any medical research (Table 3).

**Table 3:** Demographic and economic characteristics of respondents of WukiroKilte-Awulaelo and Hintal-wajirat*woredas *(districs), Tigray region, Northern Ethiopia, July 2013

Variable (*n* = 199)	Intervention f (%) (*n* = 114)	Control f (%) (*n* = 85)	Total f (%) (*n* = 199)
Age in years			
18–24	34 (29.8 %)	38 (44.7 %)	72 (36.2)
25–31	45 (39.5 %)	30 (35.3 %)	75 (37.7)
32–38	30 (26.3 %)	14 (16.5 %)	44 (22.1)
39–45	5 (4.4 %)	3 (3.5 %)	8 (4)
Mean ( ± SD)	27.9 (±6.2)	26.4 (± 6.2 )	27.3 (±6.3)
Marital status			
Married	99 (86.8 %)	67 (78.8 %)	166 (83.4)
Single	5 (4.4 %)	8 (9.4 %)	13 (6.5)
Divorced	7 (6.1 %)	6 (7.1 %)	13 (6.5)
Widowed	3 (2.6 %)	4 (4.7 %)	7 (3.5)
Educational status			
Not able to read and write	37 (32.5 %)	21 (24.7 %)	58 (29.1)
Able to read and write	11 (9.6 %)	6 (7.1 %)	17 (8.5)
Primary school	29 (25.4 %)	21 (24.7 %)	50 (25.1)
Secondary school	24 (21.1 %)	27 (31.8 %)	51 (25.6)
Diploma and above	13 (11.4 %)	10 (11.8 %)	23 (11.6)
Religion			
Orthodox Christian	100 (87.7 %)	72 (84.7 %)	172 (86.4)
Muslim	13 (9.6 %)	10 (11.8 %)	23 (11.6)
Others (Catholic/Protestant)	1 (0.9 %)	3 (3.5 %)	4 (2.0)
Mother tongue			
Tigrigna	114 (100 %)	83 (97.6 %)	197 (99)
Amharic	0	1 (1.2 %)	1 (0.5)
Others	0	1 (1.2)	1 (0.5)
Occupation			
Housewife	24 (21.1 %)	47 (55.3 %)	71 (35.7)
Farmer	38 (33.3 %)	4 (4.7 %)	42 (21.1)
Merchant	28 (24.6 %)	6 (7.1 %)	34 (17.1)
Government employee	15 (13.2 %)	13 (15.3 %)	28 (14.1)
Private employee	4 (3.5 %)	10 (11.8 %)	14 (7.0)
Others (student, jobless)	5 (4.4 %)	5 (5.9 %)	10 (5.0)
Family monthly income			
Less than 500ETB^a^	6 (5.3 %)	10 (11.8 %)	16 (8.0)
500-1000ETB	26 (22.8 %)	34 (40.0 %)	60 (30.2)
Greater than 1000ETB	82 (71.9 %)	41 (48.2 %)	123 (61.8)
Participated in medical research previously	21 (18.4 %)	15 (17.6 %)	36 (18.1)

^a^1 USD (US Dollar) ~ 19 ETB and GBP ~ 28.5 ETB

### Recruitment and retention rates

The overall study recruitment rate was 84.7 % with 88.7 % in the intervention and 80.7 % in the control groups (*p* = 0.05). The overall study retention rate was 78.3 % with 85.7 % for the intervention and 70.3 % for the control groups (*p* < 0.005). The loss-to-follow-up rates after initial consent were 14.3 % and 29.8 % in the intervention and control groups respectively.

### Informed-consent comprehension scores

In the control group, majority (77.6 %) had low comprehension score (50 % or less). Only 4.7 % had high comprehension (75 % or greater) score. The remaining 17.6 % had medium comprehension scores (between 50 % and 75 %). In the intervention group, 43 % of participants had high comprehension scores (75 % or greater), only 8.8 % had a low comprehension score (50 % or less) and the remaining 48.2 % had a medium score (between 50 % and 75 %). Participants in the intervention group were 4.6 times more likely to have medium comprehension scores and were 14.5 times more likely to have high comprehension scores than participants in the control group [Table 4]. The mean comprehension scores of the two groups (intervention and control) for all the 25 questions used in the comprehension test are presented in Table 5.

**Table 4:** Level of informed consent comprehension in the intervention and control groups, WukiroKilte-Awulaelo and Hintal-wajiratdistrics, Tigray region, Northern Ethiopia, July 2013

Comprehension Score	Intervention Group f (%) (*n* = 114)	Control Group f (%) (*n* = 85)	RR (95 % CI)	*P* value
Low [<=50 %]	10 (8.8)	66 (77.6)	1.00	
Medium [50 %-75 %]	55 (48.2)	15 (17.6)	4.6 (2.9, 7.3)	<0.0001
High [> = 75 %]	49 (43.0)	4 (4.7)	14.5 (5.6, 37.9)	<0.0001

**Table 5:** Mean informed consent comprehension score (CS) of participants, Tigray region, Northern Ethiopia, July 2013

	Consent component	Mean % CS			
		Intervention (*N* = 114)	Control (*N* = 85)	Mean difference (95 % CI)	*P* value
201	This health related study is a form of a research^a^. [True]	44.7	30.6	14.1 (0.5 , 27.8 )	0.043
202	Obligation to participate in this medical research^a^. [False]	19.3	8.2	11.1 (1.2 , 21.0)	0.029
203	Told who is funding this research^a^. [True]	46.5	34.1	12.4 (1.5 , 26.2 )	0.080
204	Told the total number of people that participate in this research^a^. [False]	57.9	44.7	13.2 (0.9, 27.3)	0.066
205	Except study team no one will be allowed to see my health information^a^. [True]	65.8	81.2	15.4 (27.9, 2.9)	0.016
206	I will be told test results from this research^b^. [False]	76.3	15.3	61.0 (49.7, 72.3)	<0.0001
207	Treated for the infection tested by this research^b^. [False]	63.2	11.8	51.4 (39.4, 63.4)	<0.0001
208	I have been told contact person address^a^. [True]	71.9	41.2	30.8 (17.5, 44.0)	<0.0001
209	I will get a special care in my regular ANC follow up^b^. [False]	64.0	55.3	8.7 (5.1, 22.6)	0.215
210	My participation in the study can be stopped at any time^a^. [True]	81.6	20.0	61.6 (5.0, 72.8)	<0.0001
211	I will be asked for costs related to my participation in this study^a^. [False]	80.7	50.6	30.1 (17.5, 42.7)	<0.0001
212	I will be paid or got any incentive for participating in this study^a^. [False]	95.6	42.4	53.3 (43.1, 63.4)	<0.0001
213	The sample taken in this study can be used for other purpose^a^. [False]	87.7	49.4	38.3 (26.6, 50.0)	<0.0001
301	Selection to participate in this study^a^. [A]	99.1	78.8	20.3 (12.5, 28.1)	<0.0001
302	When to visit a doctor to avoid cervical cancer^b^. [B]	67.5	51.8	15.8 (2.1, 29.5)	0.024
303	Who analyze and discuss test results^b^. [A]	95.6	51.8	43.3 (33.6, 54.1)	<0.0001
304	At what time can leave the study?^a^ [A]	64.0	12.9	51.1 (39.0, 63.1)	<0.0001
305	Agreed or signed to participate in this research mean^a^. [C]	87.7	61.2	26.5 (15.1, 38.0)	<0.0001
306	Can stop participation after you signed to participate?^a^ [A]	73.3	21.2	52.5 (40.4, 64.6)	<0.0001
307	Any difference made to regular ante natal care if not participate?^a^ [B]	85.1	81.2	3.9 (6.6 , 14.5)	0.466
308	The main purpose(s) of the study?^c^ To know more about cancer disease in Ethiopia [A]	67.5	56.5	11.1 (2.6 , 24.7)	0.111
	To introduce vaccine to benefit future generation of girls [B]	91.2	47.1	44.2 (33.1, 55.3)	<0.0001
309	The main benefit(s) taking part in this research?^c^ Future generation of girls but not me will benefit. [C]	69.3	36.5	32.8 (19.5, 46.2)	<0.0001
310	Procedure(s) asked to take part in?^c^ Giving a small amount blood for test [A]	96.5	65.9	30.6 (21.0, 40.3)	<0.0001
	Giving vaginal secretion for test [C]	91.2	56.5	34.8 (23.7, 45.8)	<0.0001
311	Task(s) asked to complete?^c^ Attend appointment [A]	98.2	83.5	14.7 (7.3, 22.2)	<0.0001
312	Side effect(s) that might occur during blood drawing for test?^c^ Pain or bruising on the vein [A]	57.0	41.2	15.8 (1.8, 29.9)	0.027
	Bleeding at the site of the needle [B]	47.4	34.1	13.3 (0.6 , 27.1 )	0.061
Over all C-Score		73.1	45.2	27.9 (23.96, 31.87)	<0.0001

^a^ *Generic test item- These test items appear on each version of MICCA*

^***b***^ *Trial specific test item- These test items do not appear on every version of the MICCA. They are generated based on responses to BIQ*

^c^ *Trial specific test items appear on each version of the MICCA. The response option for each of these test items are generated based on response to BIQ*

Participants in the intervention group obtained an average score of 73.1 % in overall comprehension; 73.2 % in the recall and 73.0 % in the understanding sections. Participants in the control group obtained an average score of 45.2 % in the overall comprehension; 44.8 % in the recall and 45.6 % in the understanding categories. There were statistically significant mean differences in all the three categories between the intervention and control groups (*p* < 0.001). There was a significant net difference in the mean score comprehension in almost all of the components in the intervention group compared to the standard. The highest mean difference (42.7 %) was observed in the understanding of participant rights. There was no statistically significant mean score difference in disease-related information between the intervention and control groups [Table 6].

**Table 6:** Comparison of percentage of participants giving correct responses to the main consent components in the intervention and standard group, WukiroKilte-Awulaelo and Hintal-wajirat*woredas*(districts), Tigray region, Northern Ethiopia, July 2013

Consent component	Mean % CS Intervention *N* = 114)	Mean % CS Control (*N* = 85)	Mean difference (95 % CI)	*P*
Disease information [Q302]	67.54	51.77	15.8 (2.1, 29.5)	2.4
Information about the study[Q201, 203, 204, 208]	55.26	37.64	17.6 (9.46, 25.76)	<0.001
Aim /purpose of the study[Q308A, 308B]	79.38	51.76	27.62 (19.53, 35.70)	<0.001
Selection criteria [Q301]	99.12	78.82	20.30 (12.50, 28.10)	<0.001
Procedure of sample collection [Q310A, 310C]	93.86	61.18	32.68 (25.21, 40.86)	<0.001
Tasks to be performed by participants [Q311A]	98.25	83.53	14.70 (7.30, 22.20)	<0.001
Risk of the study/ side effect of sample collection [Q312A, 312B]	52.19	37.65	14.55 (4.05, 25.04)	0.007
Benefit of the study [Q206, 207,209, 309C]	68.20	29.71	38.50 (29.38, 47.61)	<0.001
Confidentiality [Q205, 213]	76.75	65.29	11.46 (2.21, 20.71)	0.015
Participant right [Q202, 210, 212, 304, 305, 306]	70.32	27.64	42.67 (36.35, 48.99)	<0.001
Summary comprehension score				
^a^Understanding	73.01	45.61	27.40 (22.60, 32.19)	<0.001
^b^Recall	73.16	44.78	28.37 (23.34, 33.40)	<0.001
Over all C- score	73.09	45.17	27.92 (23.97, 31.87)	<0.001

^a^Understanding -score of Q201, 202, 205, 209, 210, 211, 212, 213, 301, 304, 305, 306, 307

^b^Recall- score of Q 203, 204, 206, 207, 208, 302, 303, 308A, 308B, 309C, 310A, 310C, 311A, 312A, 312B

Further analysis was done for potential socio-demographic factors associated with comprehension irrespective of REA. Participants with higher educational level (secondary and above) were found to be about 6.7 times more likely (AOR = 6.68, 95 % CI 1.01 to 44.03) to obtain high scores (> = 75 %) in the comprehension assessment than those with no formal education. Participants who had ever participated in any medical research were 2.8 times more likely to score high (AOR = 2.77, 95 % CI 1.01 to 7.58) than those who had never. Occupation and income was also found significantly associated with high score of comprehension [Table 7].

**Table 7:** Variables associated with comprehension status among study participants in WukiroKilte-awulaelo and Hintalo-wajirat districts,Tigray region, Northern Ethiopia, July 2013

Variables	Comprehension score status (*N* = 199)		AOR (95 % CI)	*P*-value
	High (> = 75 %) f(%)	Low (<75 %) f(%)		
Age in years				
18–24 yrs.	23 (34.3 %)	49 (37.1 %)	1.09 (0.38, 3.10)	0.876
25–31 yrs.	26 (38.8 %)	49 (37.1 %)	1 ( Reference gp)	
32–38 yrs.	16 (23.9 %)	28 (21.1 %)	1.7 (0.64, 4.55)	0.285
Marital status				
Married	58 (86.6 %)	108 (81.8 %)	1 (Reference gp)	
Single	3 (4.5 %)	10 (7.6 %)	1.05 (0.17, 6.58)	0.955
Divorced	4 (6.0 %)	9 (6.8 %)	0.59 (0.14, 2.53)	0.479
Religion				
Ortho. Christian	54 (80.6 %)	118 (89.4 %)	1 (Reference gp)	
Muslim	13 (19.4 %)	10 (7.6 %)	2.4 (0.80, 7.15)	0.089
Educational status				
Not able to read and write	13 (19.4 %)	45 (34.1 %)	1 (Reference gp)	
Able to read and write	6 (9.0 %)	11 (8.3 %)	2.20 (0.48, 10.15)	0.314
Within or completed primary school	15 (22.4 %)	35 (26.5 %)	2.23 (0.57, 8.72)	0.251
Within or completed secondary school	20 (29.9 %)	31 (23.5 %)	2.37 (0.52, 10.86)	0.266
Diploma and above	13 (19.4 %)	10 (7.6 %)	6.68 (1.01, 44.03)	0.048
Occupation				
House wife	8 (11.9 %)	63 (47.7 %)	1 (Reference gp)	
Private employee	4 (6.0 %)	10 (7.6 %)	3.39 (0.64, 17.92	0.151
Government employee	15 (22.4 %)	13 (9.8 %)	2.91 (0.62, 13.67)	0.177
Merchant	18 (26.9 %)	16 (12.1 %)	5.43 (1.78, 16.60)	0.003
Farmer	18 26.9 %)	24 (8.2 %)	9.05(2.79, 29.23)	0.000
Monthly family income				
= < 500 ETB	3 (4.5 %)	13 (9.8 %)	0.67 (0.13, 3.35)	0.623
>500 & < 1000 ETB	9 (13.5 %)	51 (38.6 %)	0.38 (0.15, 0.96)	0.041
> = 1000 ETB	55 (82.1 %)	68 (51.5 %)	1 (Reference gp)	
Previous participation				
Yes	21 (18.4 %)	15 (17.6 %)	2.77 (1.01, 7.58)	0.047
No	85 (74.6 %)	60 (70.6 %)	1 (Reference gp)	

### Quality of informed consent

Overall, the mean informed consent quality assessment score for the control group was 78.5 %, and that for the intervention group was 89.1 % [Table 8]. More than eighty percent (81.2 %) in the control and 99.1 % in the intervention group were satisfied with the overall consent process, while a statistically significant mean difference in the overall mean quality of informed consent scores between the intervention and control groups. Statistically significant difference was detected in all but three components of quality assessment [Table 8].

**Table 8:** Comparison of mean quality of informed consent process (QuIC) score from the participant’s perspective by intervention group in WukiroKilte-Awulaelo and Hintal-wajiratdistricts, Tigray region, Northern Ethiopia, July 2013

Informed consent process components	Quality of IC (Mean %)			
	Intervention (*n* = 114)	Control (*n* = 85)	Mean difference (95 % CI)	*P* value
There was sufficient time for consent discussion. [Agree]	93.9	82.4	11.5 (2.7, 20.3)	0.010
Agreed to participate in this study voluntarily and with full understanding. [Agree]	100	87.1	12.9 (6.7, 19.2)	<0.001
Enrolment decision made mainly by me the respondent. [Agree]	99.1	92.9	6.2 (1.0, 11.3)	0.019
Discussed about the research with other patients or participants. [Agree]	91.2	17.6	8.9 (0.5, 18.2)	0.062
Consent form read or explained carefully. [Agree]	82.5	81.2	1.3 (-9.7 , 12.2)	0.818
Consent form was important source of information. [Agree]	92.1	88.2	3.9 (-4.5 , 12.2)	0.361
Consent form was easy to understand. [Agree]	97.4	90.6	6.8 (0.4, 13.2)	0.039
Consent form was important to the decision. [Agree]	95.6	95.3	0.3 (-5.6, 6.2)	0.915
Pressure from provider to sign/agree consent form. [Disagree]	95.6	76.5	19.1 (10.1, 28.2)	<0.001
Sufficient opportunity to ask questions. [Agree]	95.6	67.1	28.6 (18.8, 38.3)	<0.001
Questions answered thoroughly by the consent provider. [Agree]	99.1	74.1	25.0 (16.6, 33.4)	<0.001
Satisfied with informed consent process. [Agree]	99.1	81.2	17.9 (10.4, 25.5)	<0.001
Decision to participate was easy or very easy. [Agree]	99.1	87.1	12.1 (5.5, 18.6)	<0.001
Mean score of Quality of informed consent	89.06	78.53	10.5 (6.8, 14.2)	<0.001

## Discussion

The study demonstrated the possible impacts of REA on study retention rate, levels of comprehension and quality of the informed consent process among study participants in a low-income setting. REA conducted prior to recruitment of participants revealed a number of relevant and context-specific ethical issues concerning the HPV study and its targeted study population. These findings helped in adapting the study information sheet, consent form and consent procedures to the local culture and educational level of the population. The HPV study was selected for the REA intervention due to expressed interest from the investigators and expressed concerns in relation to the sensitivity of the issues as the study deals with cancer of the reproductive organ and sexually transmitted infection. Such parameters which suggested that REA will be relevant as the study was expected to generate ethical issues related to the sensitivity of the subject, ambiguity of terms, and decision-making dynamics of the community. The study was conducted in a well-defined population group with relative homogeneity in language, ethno-cultural and geographic parameters. The two study sites (intervention and control) were similar to each other on major societal parameters. They were geographically adjacent to each other and belonged to the same ethno-cultural cosmos including the health system. The socio-demographic characteristics of study participants in both locations revealed overall similarity except few irregularities. We employed MICCA and BIQ for measuring comprehension and QuIC for assessing quality of informed consent. The tools were able to capture both standard informed consent items as well as study-specific test items [17]. Certain allowable practical adjustments were done on the tools and some of the components, otherwise both tools have been validated for use by researchers in different settings [17].

Recruitment rate and the recruitment index are considered important parameters in monitoring the quality of research implementation [6, 7]. In this study higher rates of both recruitment and retention were documented for the intervention group. While we documented strong statistical association between study retention rate and use of REA-based revision on informed consent process, no statistically significant difference was documented between the recruitment rates of the two groups. Previous studies have tried to identify potential contributors to recruitment rates of study participant such as 'opt-out' approaches [20], early consideration of participants' perspectives [21], addressing ethical issues such as the therapeutic misconception [6], and establishing trust between researchers and potential participants [22]. The recruitment rate, however, may have been affected by other factors beyond the consent information as the complexities of the challenges of recruitment are also determined by the complexities of the respective studies [7, 23, 24] and decisions being made prior to recruitment based on information circulating in the community [25].

Recruitment rate has been documented as an important determinant of subsequent retention [6, 24]. Other factors affecting retention include mental state of the person [26], availability of tracking and follow-up mechanisms [27], availability of mechanisms addressing community and context-specific issues and needs in special communities [28]. However every study has its own peculiar features and it may be difficult to generalise these factors. The registered improvement in the study retention rate in our study may have resulted from improved understanding of the consent information.

The current study documented significant differences in the comprehension levels (understanding and recall) between the intervention and control groups. REA-based adjustments to the content and delivery of consent information in the intervention group were linked to the improved levels of understanding and recall in the intervention group. These findings concur with studies which have reported improved consent information comprehension with appropriate interventions such as provision of more explanation and socio-culturally tailored approach [29]. Provision of tailored terminologies and additional narrative explanations on the pre-identified ethical issues and allowing more time for discussion and questions from the participants in the intervention group might have been potential determinants. There was no significant difference in comprehension levels related to disease information between the intervention and control group. The reasons for non-significance could be the sensitivity and novelty of the issues of cervical cancer in the area.

In the study there was a significant difference in the overall levels of perceived quality of the consent process in the intervention compared to the control group. There was a statistically significant difference in all but three of the components of quality assessment; whether consent form was read and explained carefully; whether consent from was important source of information; and whether consent form was important for consent decision. Possible reasons for the improved perception of the quality of the consent process in this study include the improvements made in the consent information and the revised consent procedures both of which were geared to the needs and concerns of the potential participants, based on the REA. There are several possible reasons for lack of statistically significant improvements in three areas. Regarding readability, the participants in the intervention groups mainly had the form read to them rather than reading it themselves. Another reason is that all three measures already scored highly in the control group with little room for improvements. Though not significant, there were improvements observed in the three categories and if a larger sample had been used this might have been significant. Quality of informed consent depends on factors such as type of consent information provided, amount of information, adequacy of comprehension, and the voluntariness of decision making by the participants [5]. In the clinical set up, participant-related factors such as age, IQ, level of cognitive function and external locus of control were associated with poor quality and information recall. Written information provided immediately before admission was associated with better outcomes [30].

Various researchers have utilized different interventions to improve the consent process and understanding, such as use of local narratives [31], extended one-to-one discussion [32–34], enhanced consent [33, 35], a revised consent process [36], and a booklet of participants’ rights [37]. Cultural and linguistic modifications to the informed consent process were considered to significantly enhance understanding by study participant in low income settings [29]. On the other hand it has been shown that lexicosyntactic readability improvement of the consent form [38], multimedia enhanced consent [34], and use of a concise version of the consent form [39] do not have effects on comprehension. There are still ongoing evidence-based debates as to which interventions are effective in enhancing comprehension [40]. The intervention in this study involved improvements changes in the consent procedures based on REA findings.

One of the ethical dilemmas faced by the researchers while revising the consent form is what level of change on an IRB-approved form would be appropriate and allowable once in the field, and what principles should govern this. According to existing guidelines, any amendments of an approved research project require further IRB approval before implementation [41, 42]. The only exceptions to this are if the changes are immediately and urgently required for the safety of the subjects. In this case, the IRB must be informed immediately after establishing the need for the changes -in most cases within five business days [43]. The other possible exception to this rule is if the study is a determined to be non-research by the IRB and does not need further IRB follow-up [41]. We made revisions to the consent form in the field on the following basis; a) that the revision added to the approved consent and did not reduce any major component (the consent form content and structure approved by IRB were maintained with improvements); b) that the comprehension assessment project and the possibility of revising the consent form were already approved by IRB; c) that the changes were important for the welfare of the subjects in the study. Since getting approval from IRB for the modification might take time, some provision for making appropriate adjustment without tampering with the basic components of consent may be necessary in future.

The Ottawa Statement provides a framework for addressing ethical issues around cluster randomised trials. According to the statement on the Ethical Design and Conduct of Cluster Randomized Trials, "When participants’ informed consent is required, but recruitment of participants is not possible before randomization of clusters, researchers must seek participants’ consent for trial enrollment as soon as possible after cluster randomization—that is, as soon as the potential participant has been identified, but before the participant has undergone any study interventions or data collection procedures" [44, 45]. In the current study consent was obtained soon after recruitment in to the planned study without further additional approval of the REA based revisions on the originally approved consent form, based on the premises presented above.

The study has some potential limitations. Efforts were made to ensure the uniformity of the intervention and control sites, however there may have been undocumented differences in the settings which may have confounded the study outcomes. Both levels of comprehension and quality of the informed consent process are based on participant perceptions using questionnaire based interviews which were not accompanied by independent observation methods. This might reduce the objectivity and validity of the responses. Reasons for loss to follow-up of those who did not attend were not documented, and neither did we document the basic socio-demographic characteristics of non-attendees. The sample size determination was done based on assumptions from other countries as there was no local estimate available. In addition we assumed a 10 % non-response rate while the non-response rate for the study was about 15 %. The single cluster intervention also might have reduced the study power.

## Conclusions

Based on our findings, REA is associated with improved understanding of study concepts, purposes and procedures and participants’ perceptions of the informed consent process. Investing in an intervention which enhances researchers' understanding of the local context and tailoring the consent information and process is associated with improvement s in overall consent comprehension, study recruitment rates and retention rates in low-income settings. REA is also an appropriate intervention for further use in similar settings and situations.

We would like to encourage researchers and research ethics committees and policy makers to employ REA to improve research consent comprehension, recruitment and retention levels in certain low income settings. As this is the first study exploring the effects of REA on comprehension, additional studies in other settings with more objective methods of measurement would provide greater insight into the overall effects of REA on comprehension of consent. Such studies might even expand into consent processes for medical interventions. Ethicists and research policy makers are encouraged to explore the consequences of utilizing REA in the light of IRB requirements and operating procedures. In future studies, if REA is planned, it should be included in the research proposal, so it and any subsequent consent form modification are approved by the IRB.

## Abbreviations

ANC, Ante natal care; BIQ, Brief investigator questionnaire; HPV, Human papilloma virus; IRB, Institutional review board; MICCA, Modular informed consent comprehension assessment; QuIC, Quality of informed consent; REA, Rapid ethical assessment; SPSS, Statistical package for social sciences

## Additional files

Additional file 1: Standard information sheet and Consent form for “HPV-Subtype Prevalence Study”. This version is the non-modified information sheet and consent form, the version initially approved by the IRB. This is the very version of information sheet and consent form administered used the control group. (DOCX 17 kb) Additional file 2: Modified information sheet and consent form for ''HPV-Subtype Prevalence Study''. This is the modified version of information sheet and consent form administered for the intervention group. Modifications were done on the IRB approved version based on the REA (Rapid Ethical Assessment) conducted prior recruitment. (DOCX 21 kb) Additional file 3: Data collection materials used for comprehension assessment in "HPV-subtypes Prevalence Study". These are the data collection material used for testing comprehension and quality of informed consent in both the control and intervention groups. The material were developed based on Modular Informed Consent Comprehension Assessment (MICCA), Brief Investigator Questionnaire (BIQ) and Quality of Informed Consent (QuIC) tools. (DOCX 28 kb)
