# Supplementary material for: Cluster randomized trial assessing the effects of rapid ethical assessment on informed consent comprehension in a low-resource setting
Source: BMC Med Ethics. 2016 Jul 12;17:40. doi: 10.1186/s12910-016-0127-z (PMC4943010; doi:10.1186/s12910-016-0127-z)
Supplement: Additional file 1: — Standard information sheet and Consent form for “HPV-Subtype Prevalence Study”. This version is the non-modified information sheet and consent form, the version initially approved by the IRB. This is the very version of information sheet and consent form administered used the control group. (DOCX 17 kb) [file 12910_2016_127_MOESM1_ESM.docx]

Additional file 1: Standard information sheet and Consent form for “HPV-Subtype Prevalence Study”

**Information sheet**

**Introduction**: Good morning, my name is _____________.I am part of a group from Addis Ababa School of Public Health. We are trying to find out more about cancer diseases in Ethiopian women. Most common is cervical cancer. Sometimes infections like HPV and others can make the cervix weak and then a cervical cancer can grow. There are different options to help women to avoid cervical cancer. Please always see a doctor if there is too much vaginal bleeding. To implement a vaccination in the future, information on HPV infections and co-infections must be collected. We want to collect information in 600 women from different parts of Ethiopia. We ask you if you are willing to give a small drop of blood (when you are in the lab) and if you could take a small amount of fluid from the vagina for us. This does not cause any harm to you or to the baby. Someone will assist you to do that.

There will be no names on the blood and fluid sample – they will be anonymous. Therefore information on the results will not be given back to you. The samples will be analysed and doctors from School of Public Health at Addis Ababa University will discuss the results. Then the government can decide to implement a vaccination against cervical cancer in the country. The future generation of girls will benefit.

**Risk and Benefits**: There is no perceived harm to the individual participants. To maintain privacy and confidentiality, all the specimen (body fluid and blood) will be linked and stored totally anonymous. The data will be kept in password and all formats will be properly locked in. Since the investigations are for epidemiological research, high-throughput testing is done. The findings of the survey will inform further policy related discussions for cervical cancer in Ethiopia. The infections tested are naturally acquired infections. Detection of prevalence of viruses will help subsequently to implement national programmes for immunisation.

**Study Consent and Decision to Participate**: Informed consent will be taken from each person in advance to every interview. Participation is purely on voluntary bases. You are free to decide to take part in the study or not. If you do not want to answer any of the questions you have the right not to answer. You can stop the interview any time as well. The names of the interviewees will be kept in a separate code list until all data is clarified and there is no need to come back to the interviewee. All personnel data will be kept in a separate list with codes. Results of the interviews will be recorded with the code. After the end of the interview-phase, all codes will be destroyed.We would be very happy if you could help us in this study. But you can decide not to take part in the study and this will not in any way interfere with your ANC clinic follow-up. Note that your regular antenatal clinic will be done in any case – if you decide to participate or if you do not participate in the study.

**Contact Persons for any question:**

**Investigator’s Address**

Dr Adamu Addissie, AAU- SPH, Addis Ababa

Tel – 01155473xx

**IRB Contact**

Dr Yimtubezinash W/Amanuel, AAU, CHS-IRB, Addis Ababa

Tel – 0118961xx

**Consent Form**

I __________________ after understanding the purpose of the study understanding that I have the right to take part or to withdraw from the study have decided to take part in the study.

I am aware that the study is carried out by medical doctors in order to find out more about women 18-45 years of age in the area and also authorities are aware of the study carried out by physicians from the local, Addis Ababa University and collaborators. I am aware that the researchers would like to ask about reproductive health issues in women and will take blood and vaginal specimens for testing.

I have also understood that all information given will be confidential – no personnel data will be revealed to the public. After completion of the study, all names will be deleted I hereby sign to express my consent to take part in the study out of my voluntary will.

If Agree Sign of participant ________Disagree________________( refusal )

(For data collectors: use explanation in the bottom to appoint after 2 weeks)

Signature of the data collector _____________ date _________________ code: ________

**Appointment after two weeks:** *Thank you for participation in this study. Today there will no blood or vaginal secretion taking. We ask you again if you are voluntarily to come back after* ***two*** *weeks for second part interview of this study and we will discuss about giving the sample then*. [**For data collector:** *give an appointment about* ***12-16 days*** *after first consent taking. The date should be at their convenience.]*
