# Supplementary material for: Cluster randomized trial assessing the effects of rapid ethical assessment on informed consent comprehension in a low-resource setting
Source: BMC Med Ethics. 2016 Jul 12;17:40. doi: 10.1186/s12910-016-0127-z (PMC4943010; doi:10.1186/s12910-016-0127-z)
Supplement: Additional file 2: — Modified information sheet and consent form for ''HPV-Subtype Prevalence Study''. This is the modified version of information sheet and consent form administered for the intervention group. Modifications were done on the IRB approved version based on the REA (Rapid Ethical Assessment) conducted prior recruitment. (DOCX 21 kb) [file 12910_2016_127_MOESM2_ESM.docx]

**Additional file 2: Modified information sheet and consent form for ''HPV-Subtype Prevalence Study''**

**Information sheet**

**Introduction**: Hello /Greetings, my name is _____________. I am health professional. I am member of a team from Addis Ababa University School of Public Health. We are trying to find out more about cancer (Menkersa or Menshiro) diseases in Ethiopian women. Most common is cervical cancer. Sometimes infections like “Human Papiloma Virus“ and other infections can make the cervix weak and then a cervical cancer can grow. There are different options to help women to avoid cervical cancer. **Please** always see a doctor if there is too much vaginal bleeding. Vaccination for human papiloma virus (virus that cause infection of the cervix) is one possible option to prevent cervical cancer.

To implement a vaccination in the future, information on HPV infections and co-infections must be collected. We want to collect information in 600 women from different parts of Ethiopia. From this study area we need 75 volunteer pregnant women. This study conducted by Addis Ababa University in coorporation with Saale University of Germany. The study is approved by respective authority at different levels and the administrative office of this district. This study has no any hidden political or religious agenda.

**Procedures**: The study process includes a face to face interview, drawing a small amount of blood and small vaginal fluid (secretion) from pregnant mothers. Pregnant mothers selected by chance when they come for their regular ante natal care follow up. The sample will be taken by a female health professional. The collected samples will be analysed and doctors from School of Public Health at Addis Ababa University will discuss on the results. Then the findings help the government to implement vaccine against cervical cancer in the future.

**Risk and Benefits**: There is no perceived harm to you or your baby. However, you may feel some pain, bruise or minimal bleeding as a result of the injection. You may also feel discomfort following disclosing your private parts.The result of the HPV-test will not be given back to you because there is no treatment for human papilloma virus. This infection is naturally acquired. The benefit will only be for future generations. The findings of the survey will inform further policy related discussions for cervical cancer in Ethiopia. Detection of prevalence of the virus will subsequently help the government to implement national programmes for immunisation against cervical cancer.

**Study Consent and Decision to Participate**: - Participation is purely on voluntary bases. You can decide not to take part in the study and this will not in any way interfere with your ante natal care follow-up. If you do not want to answer any of the questions you have the right not to answer. You can also stop the interview at any time.

To maintain privacy and confidentiality, there will be no names on the questiannare, blood and vaginal fluid (secretion) sample. We use codes instead. Therefore information on the results will not be given back to you.No personnel data will be revealed to the public. The collected data and samples will not be used other than for the purpose of this study.

**Contact Persons for any question about the study:**

**Investigator’s Address**

*Dr Adamu Addissie, AAU- SPH, Addis Ababa*

*Tel – 01155473XX*

**Ithical review board Contact**

*Dr Yimtubezinash W/Amanuel, AAU, CHS-IRB, Addis Ababa*

*Tel – 01189613XX*

**Consent Form**

Now I can continue the interview if and only if you are volunteer to participate. As I explain you before, if you agree to participate in this study you are expected to respond to a questionnaire, give asmall amout of blood and vaginal secretion. A female health professional will assist you to do that. If you believe that you have clear and enough information about the study, you are kindly invited to participate in this study. I would like to remind you again that you have the right not to take part or to withdraw from the study at any time. However, your participation has a great contribution for the success of this study.

**Note that** your regular antenatal clinic will be done in any case – weather you decide or not to participate in this study.

In general do you have any question or concern about the study?

1. Yes (**for data collector**: give enough explanation for questions and concerns, use Annex II)
2. No (**for data collector**: ask for consent)

Are you willing to participate? (**For data collector**: tick in the box)

1. Yes, I am will to participate

(**For data collectors:** *Thank for participation and appoint for second part interview of this study. Use explanation in the bottom*)

1. No, I am not willing to participate

(Thank the participant and end the interview)

I ______________________ [the data collector] assure that I have explain all necessary information about this study to the respondent and she agree to participate voluntarily.

Signature of the data collector _______________ date ___________________ code: ________

**Appointment after two weeks:** *Thank you for participation in this study. Today there will no blood or vaginal secretion taking. We ask you again if you are voluntarily to come back after* ***two*** *weeks for second part interview of this study and we will discuss about giving the sample then*. [**For data collector:** *give an appointment about* ***12-16 days*** *after first consent taking. The date should be at their convenience.]*

Annex II: **Narrative Explanations used in the intervention group**

1. **Research** is a work trying to find new facts. For example a farmer wants to use new method of farming like sowing in line or to use fertilizer to make his farm land more productive. So first he tries the new method or the fertilizer in a plot of land. If the product is better enough then he use this method for the Whole of his farm land. This best experience will be expanded to his neighbors.
2. **Medical research:** In a medical research is also the same. If the findings from few study subjects are important for health, then will be used for the majority of the community. As you know before many years there was no vaccine for measles (Nifyo, local language). Many children were blind and died due to this disease. Now after research has been done vaccine for measles has found and many children saved from blindness and death by vaccination. Similarly this study is to introduce vaccine against “Human Papilloma Virus” and to prevent cervical cancer. Then future generation girls could be benefited.
3. **Use local words:** for cancer**: - “menkersa” (very** common**), cancer (some**) **“menshiro” (**rarely).
