# Supplementary material for: Cluster randomized trial assessing the effects of rapid ethical assessment on informed consent comprehension in a low-resource setting
Source: BMC Med Ethics. 2016 Jul 12;17:40. doi: 10.1186/s12910-016-0127-z (PMC4943010; doi:10.1186/s12910-016-0127-z)
Supplement: Additional file 3: — Data collection materials used for comprehension assessment in "HPV-subtypes Prevalence Study". These are the data collection material used for testing comprehension and quality of informed consent in both the control and intervention groups. The material were developed based on Modular Informed Consent Comprehension Assessment (MICCA), Brief Investigator Questionnaire (BIQ) and Quality of Informed Consent (QuIC) tools. (DOCX 28 kb) [file 12910_2016_127_MOESM3_ESM.docx]

**Additional file 3: Data collection materials used for comprehension assessment in "HPV-subtypes Prevalence Study"**

**PART-I: SOCIO DEMOGRAPHIC DATA OF PARTICIPANTS**

| **S. No** | **Question** | **Response and Coding** |
| --- | --- | --- |
| 101 | Age (in complete years) | ________years |
| 102 | What is your current marital status? | Single …………………………...…1  Married ……………………................2  Divorced …………………….…..…..3  Widowed……………………….…...4 |
| 103 | What is your highest educational status? | Cannot read and write…..….……...…1  Can read and write ………….…..........2  Grade 1-8 completed ………….……..3  Grade 9-12 completed .………..…….4  Diploma and above ………………..5 |
| 104 | What is your religion? | Orthodox Christian ………………...1  Muslim ………………………..........2  Others (specify) 88 |
| 105 | What is your ethnicity? | Tigre…………………………….…..1  Amhara…………………………...…2  Others (specify)88 |
| 106 | What is your mother tongue or commonly used language? | Tigrigna ………………………..…..1  Amharic………………………..…..2  Others (specify)88 |
| 107 | What is your current occupation? | Housewife……………………..….1  Private employee……………….…2  Government employee……………3  Daily laborer………………………4  Merchant………………………….5  Farmer …………………………....6  Others (specify) ……………..…..88 |
| 108 | What is your family total monthly income? ETB | < 500 ETB…………………....….1  500-1000 ETB…………….…….2  >1000ETB……………….…..….3 |
| 109 | Have you ever participated in any medical research? | Yes ……………………….……….1  No ………………………….……..2  I don’t know …………………….99 |

**PART II: INFORMED CONSENT COMPREHENSION ASSESSMENT TEST**

**Instruction 1**: please circle the numbers that best answers each statement listed below. (**You may only circle on one number for each question)**.

| **S.No** | **Question** | **True** | **False** | **I do not know/ I am not sure** |
| --- | --- | --- | --- | --- |
| 201 | This health related study is a form of a research^1^. | 1 | 2 | 99 |
| 202 | It is my obligation to participate in this medical research^1^. | 1 | 2 | 99 |
| 203 | I have been told who is funding this research^1^. | 1 | 2 | 99 |
| 204 | I have been told the total number of people that participate in this research^1^. | 1 | 2 | 99 |
| 205 | During this research other than the study team no one will be allowed to see my health information^1^. | 1 | 2 | 99 |
| 206 | I will be told about my test results from this research^2^. | 1 | 2 | 99 |
| 207 | I will be treated for the infection tested by this research^2^. | 1 | 2 | 99 |
| 208 | I have been given the name and phone number of the person to contact if I have questions or concerns about the research^1^. | 1 | 2 | 99 |
| 209 | I will get a special care in my regular ante natal care in response to my participation in this research ^2^_._ | 1 | 2 | 99 |
| 210 | My participation in the study can be stopped at any time without any form of prejudice^1^. | 1 | 2 | 99 |
| 211 | I will be asked for costs related to participating in this study^1^. | 1 | 2 | 99 |
| 212 | I will be paid or got any incentive for participating in this study^1^. | 1 | 2 | 99 |
| 213 | The sample taken from me can be used other than the purpose of this study^1^. | 1 | 2 | 99 |

_________________________________________________________________________________________________________________________________

**^1^**Generic test item- These test items appear on each version of MICCA

**^2^**Trial specific test item- These test items are not appears on every version of the MICCA. They are generated based on response to BIQ.

**Instruction 2**: for question **301-307**, please circle the numbers that best answers each statement listed below. (**You may only circle on one number for each question**.)

1. How do you selected to participate in this study? **^2^**
2. I was asked to participate in this study when I come for regular my Ante Natal Care follow up.
3. I was selected to participate in this study based on my health situation and suspecting that I might have cancer
4. I don’t know how I was selected
5. When do you have to visit your doctor to avoid cervical cancer? **^2^**
6. Always
7. When I have too much vaginal bleeding
8. I don’t know
9. Who analyze and discuss your test results? **^2^**
10. Doctors from Addis Ababa University
11. My routine Ante natal care providers
12. I don’t know
13. At what time can you leave the study?**^1^**
14. I can leave at any time
15. I can only leave if the investigator is volunteer
16. I can only leave after all data has been collected
17. I don’t know
18. What does it mean when you agreed or signed to participate in this research?**^1^**
19. I will be legally asked if not participate in this research
20. My participation will be obligatory
21. I agreed voluntarily to participate in this study
22. I don’t know
23. Can you leave this study if you want to stop after you singed to participate?**^1^**
24. Yes
25. No
26. I don’t know
27. Suppose that you had decided not to participate in this study, do you think that would have made any difference to your regular ante natal care?**^1^**
28. Yes
29. No
30. I don’t know

**Instructions 3:-** For questions **308-313** you may circle on **more than one** number for each question.

1. Which describes the main purpose(s) of the study?**^3^**
2. To know more about cancer disease in Ethiopia
3. To introduce vaccine which benefit future generation girls
4. To improve my own medical/health condition
5. I don’t know
6. Which describes the main benefit(s) taking part in this research? **^3^**
7. I will be informed of my test results
8. I will be treated according to my test results
9. Future generation girls but not me will be benefited
10. I don’t know
11. Which procedure(s) you asked to take part in? **^3^**
12. Giving a small amount blood for test
13. Having X-ray examination
14. Giving vaginal secretion for test
15. I don’t know
16. Which task(s) will be asked to complete? **^3^**
17. Attend on your appointment
18. Coming without eating food
19. I don’t know
20. Which side effect(s) might occur during blood drawing for test?**^3^**
21. Pain or bruising on the vein
22. Bleeding at the site of the needle
23. It cause blood deficiency
24. I don’t know
25. What is your concern or fear taking part in this study?**^3^**
26. This study may have hidden religious or political agenda
27. I might be diagnosed to have cancer in this study
28. Other people may know my test result
29. I have no any concern or fear

­­_________________________________________________________________________

**^1^**Generic test item- These test items appear on each version of MICCA

**^2^**Trial specific test item- These test items are not appears on every version of the MICCA. They are generated based on response to BIQ.

**^3^**Trial specific test items appear on each version of the MICCA. The response option for each of these test items are generated based on response to BIQ.

**Instruction 4:** Please tell us (circle the number) how often you use the following resources to gather health information. **Please circle only one number for each item.**

| S.No | **Source** | **Always** | **Sometimes** | **Never** |
| --- | --- | --- | --- | --- |
| 401 | Books/Journals | **1** | **2** | **3** |
| 402 | Friends/Families | **1** | **2** | **3** |
| 403 | Health care provider (Drs, nurses, health extension professionals etc.) | **1** | **2** | **3** |
| 404 | Internet | **1** | **2** | **3** |
| 405 | Popular magazines | **1** | **2** | **3** |
| 406 | Radio | **1** | **2** | **3** |
| 407 | TV/Movies | **1** | **2** | **3** |

**PART III: Quality of Informed Consent (QuIC) process assessment**

**Instruction:** Please tell us (circle the numbers below) how the information given to you and your decision making was to involve in this study. (**Please circle only one number for each item.)** When you respond to these questions feel free that the questions are just to know the situation of agreement and how do you understand about the study you agreed to participate. **Not** to measure you general knowledge.

| **S No** | **Questions** | **Agree** | **disagree** | **I don’t know**  **/I am not sure/** |
| --- | --- | --- | --- | --- |
| 501 | There was sufficient time for consent discussion | **1** | **2** | **99** |
| 502 | Agreed to participate in this study voluntary and with full understanding | **1** | **2** | **99** |
| 503 | Enrolment decision made mainly by me the respondent | **1** | **2** | **99** |
| 504 | Discussed about the research with other patients or participants | **1** | **2** | **99** |
| 505 | Consent form read or explained carefully | **1** | **2** | **99** |
| 506 | Consent form was important source of information | **1** | **2** | **99** |
| 507 | Consent form was easy to understand | **1** | **2** | **99** |
| 508 | Consent form was important to the decision | **1** | **2** | **99** |
| 509 | Pressure from provider to sign/agree/ consent form | **1** | **2** | **99** |
| 510 | Sufficient opportunity to ask questions | **1** | **2** | **99** |
| 511 | Questions answered thoroughly by the consent provider | **1** | **2** | **99** |
| 512 | Satisfied with informed consent process | **1** | **2** | **99** |
| 513 | Decision to participate was easy or very easy | **1** | **2** | **99** |
